# Comparison and combination of mutation and methylation-based urine tests for bladder cancer detection

**DOI:** 10.1186/s40364-024-00682-x

**Published:** 2024-11-07

**Authors:** Naheema S. Gordon, Elspeth K. McGuigan, Michaela Ondasova, Jennifer Knight, Laura A. Baxter, Sascha Ott, Robert K. Hastings, Maurice P. Zeegers, Nicholas D. James, K. K. Cheng, Anshita Goel, Minghao Yu, Roland Arnold, Richard T. Bryan, Douglas G. Ward

**Affiliations:** 1 The Bladder Cancer Research Centre, Department of Cancer and Genomic Sciences, College of Medicine and Health, University of Birmingham, Birmingham, B15 2TT UK; 2 Bioinformatics & Digital Health Services, Research Technology Platforms, University of Warwick, Coventry, CV4 7AL UK; 3 Nonacus Ltd, Unit 5, Quinton Business Park, Birmingham, B32 1AF UK; 4 Department of Epidemiology, School of Nutrition and Translational Research in Metabolism, University of Maastricht, Maastricht, The Netherlands; 5 The Institute of Cancer Research, London, SM2 5NG UK; 6 Department of Applied health Sciences, College of Medicine and Health, University of Birmingham, Birmingham, B15 2TT UK

**Keywords:** Bladder cancer, Urine test, Biomarker, Mutation, Methylation

## Abstract

**Background and aims:**

Several non-invasive tests for detecting bladder cancer (BC) are commercially available and are based on detecting small panels of BC-associated mutations and/or methylation changes in urine DNA. However, it is not clear which type of biomarker is best, or if a combination of the two is needed. In this study we address this question by taking a 23-gene mutation panel (GALEAS™ Bladder, GB) and testing if adding a panel of methylation markers improves the sensitivity of BC detection.

**Methods:**

Twenty-three methylation markers were assessed in urine DNA by bisulphite conversion, multiplex PCR, and next generation sequencing in 118 randomly selected haematuria patients with pre-existing GB data (56 BCs and 62 non-BCs), split into training and test sets. We also analysed an additional 16 GB false-negative urine DNAs.

**Results:**

The methylation panel detected bladder cancer in haematuria patients with 69% sensitivity at 96% specificity (test set results, 95% CIs 52-87% and 80-99%, respectively). Corresponding sensitivity and specificity for GB were 92% and 89%. Methylation and mutation markers were highly concordant in urine, with all GB false-negative samples also negative for methylation markers.

**Conclusions and limitations:**

Our data show that, with a comprehensive mutation panel, any gains from adding methylation markers are, at best, marginal. It is likely that low tumour content is the commonest cause of false-negative urine test results. Our study does have a limited sample size and other methylation markers might behave differently to the those studied here.

**Supplementary Information:**

The online version contains supplementary material available at 10.1186/s40364-024-00682-x.

## To the editor

The principal modality for bladder cancer detection for both initial diagnosis and surveillance is flexible cystoscopy [1]. Flexible cystoscopy is invasive, inconvenient and expensive and has notable false-positive and false-negative rates [2]. Bladder tumours are in prolonged direct contact with urine and much research has focussed on urine biomarkers as an addition or alternative to cystoscopy with both RNA-based and DNA-based tests now in the marketplace. A combination of inadequate validation and significant numbers of false-positive and false-negative results hinder widespread clinical uptake.

Commercially-available urine DNA-based BC tests include AssureMDx [3] (*OTX1*,* ONECUT2* and *TWIST1* methylation + *FGFR3*,* TERT* and *HRAS* mutations), BladMetrix [4] (ddPCR, 8 methylation markers), Epicheck [5] (qPCR, 15 methylation markers), GALEAS™ Bladder [6] (NGS, mutations in 23 genes), UroDiag [7] (qPCR, *FGFR3* mutations + *HS3ST2*,* SEPTIN9* and *SLIT2* methylation), UriFind [8] (2 methylation markers) and Uromonitor-V2 [9] (qPCR, *TERT*,* FGFR3* and *KRAS* mutations). Most of these competing solutions measure a small number of biomarkers and it is logical to predict that adding more mutations or methylation markers could improve sensitivity. GALEAS Bladder (GB) is the only NGS-based method with large-scale validation that detects a comprehensive panel of BC mutations even if present at very low levels in urine DNA [6]. It is unknown if adding methylation markers to a mutation panel of this nature will improve sensitivity. This might be the case if, for example, methylation changes extend beyond the tumour itself into a field effect.

Here we investigate whether the addition of methylation markers to GB could improve test sensitivity, thereby reducing the number of false-negative results and increasing patient and clinician acceptance. We selected 23 methylation markers from the literature, The Cancer Genome Atlas and in-house data (Table S1), and developed an assay compatible with the GB workflow based on bisulphite conversion, multiplex PCR, adapter ligation and deep sequencing. The methylation assay was applied to urine cell pellet DNA from 134 haematuria clinic urines (patient information and experimental and data analysis methods are presented in Supplemental Information).

GB is an error-supressed deep-sequencing BC test based on detecting mutations in 23 BC-associated genes (https://nonacus.com/oncology/galeas-bladder-cancer-test/); the training and test set data have previously been reported [6]. The 16 false-negative samples were selected from an additional 211 BC patient urine samples (unpublished data). Variant calling and test results (positive/negative) used the GB proprietary bioinformatic pipeline.

**Fig. 1:**
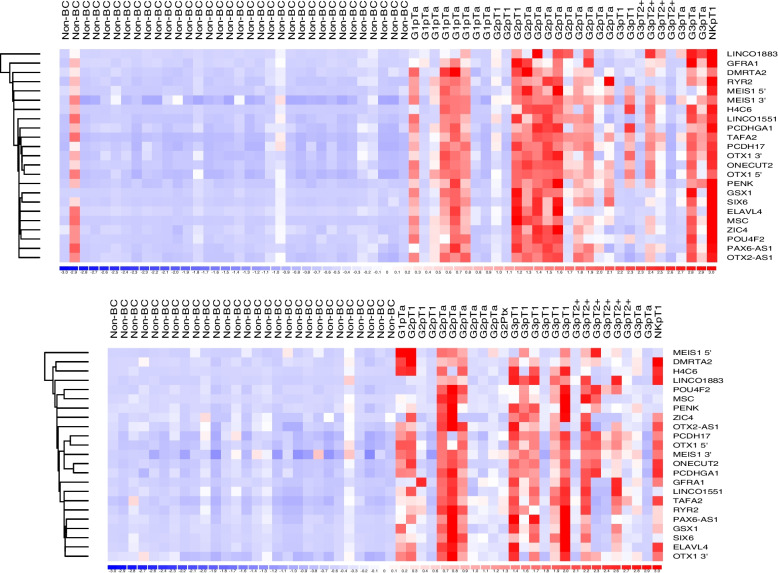
Urine DNA methylation in haematuria patients with/without bladder cancer. The training data are shown in the upper heatmap and the testing data in the lower panel. Methylation marker genomic coordinates and primer sequences are available in Table S4

All markers in the methylation panel were significantly hypermethylated in BC patient urines compared to non-BC patients (all *p* < 1 × 10^−6^, Fig. 1 and Table S3). There were no statistically significant methylation differences between BC stages and grades. In the training set, positive methylation results were obtained for 22 out of 30 cancer patients and 2 out of 34 non-cancer controls (73% sensitivity at 94% specificity). In an independent test set of urines from 54 patients (28 with BC) methylation demonstrated 69% sensitivity at 96% specificity.

**Fig. 2:**
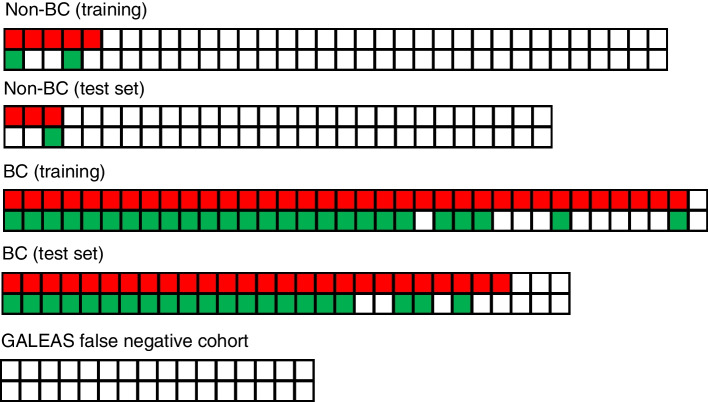
Concordance between GALEAS Bladder and methylation results. For each plot the top row is the GALEAS Bladder result and the lower row the methylation result and each column represents one patient (red = positive GB result, green = positive methylation result, white = negative result)

GB provided 93% sensitivity at 85% specificity (training set) and 92% sensitivity at 89% specificity (test set); 93% sensitivity at 87% specificity when combined. Concordance between GB and methylation results (positive/negative) was 85% (Fig. 2) with most of the discrepancies due to the lower sensitivity of the methylation test. Methylation levels also strongly correlated with variant allele frequencies in the GB mutation data (Figure S1). Of 20 GB false-negatives, none were positive for methylation markers suggesting no benefit to running both tests.

The GB panel is small enough to avoid false positives due to clonal haematopoiesis and polymorphisms, and sequencing errors are highly supressed by using unique molecular identifiers [6]. Hence, GB false-positives may be due to premalignant changes or non-visible tumours. The GB panel is comprehensive enough to detect mutations in 96% of incident bladder tumour tissues [10]. GB false-negatives could arise because tumours are not shedding cells into the urine and/or because shed cells do not contain mutations within the panel. The current study tested which of these phenomena is responsible for false-negative GB results: if a lack of mutations was responsible then, with the methylation panel exhibiting 70% sensitivity, we would expect 14 of the 20 GB false negative urine samples to be positive for our methylation panel. This was not the case; all of the samples giving false-negative GB results also gave false-negative methylation results. We conclude that mutations and methylation changes likely occur within the same population of cells and it is the low number of these cells in some urine cell pellets which is the main cause of false-negative results. We conclude that GB reliably detects BC DNA when it is present in urine, and that methylation analyses are unlikely to overcome the problem of low tumour cell fraction in urine, particularly in low-grade low-stage tumours (see Table S2).

In summary, the levels of SNVs and methylation markers in urine DNA are highly correlated such that in most samples where mutations are absent or below the limit of detection, methylation markers are also absent. It seems likely that with current approaches it will be difficult to increase sensitivity and specificity much beyond 90–95% in unselected haematuria clinic urine samples, although we cannot exclude the possibilities that optimising preanalytical factors or analysing complex (epi)genome-wide profiles could lead to increased test accuracy.

## Supplementary Information

Supplementary Material 1.

## Data Availability

Data available upon reasonable request to the corresponding author.
