# Supplementary material for: Comparison and combination of mutation and methylation-based urine tests for bladder cancer detection
Source: Biomark Res. 2024 Nov 7;12:133. doi: 10.1186/s40364-024-00682-x (PMC11542462; doi:10.1186/s40364-024-00682-x)
Supplement: Supplementary file 1 — Supplementary Material 1. [file 40364_2024_682_MOESM1_ESM.docx]

**Supplemental Information**

Patients and samples

Urine was collected from patients in the multi-centre Bladder Cancer Prognosis Programme (BCPP, UK ethical approval 06/MRE04/65, East Midlands - Derby - Ethics Committee) and from haematuria clinics at The Queen Elizabeth Hospital, Birmingham, UK (UK ethical approval 15/NW/0079, North West – Haydock - Research Ethics Committee) [1]. All patients provided informed written consent for sample and data collection for biomarker research. Up to 50mls of urine were collected in plain centrifuge tubes, transported on ice, and centrifuged at 1000g for 10 minutes; cell pellets were then separated and frozen at -80°C on the same day. DNA was extracted from cell pellets using the Quick-DNA Urine Kit (Zymo Research D3061) and quantitated using the high-sensitivity dsDNA Qubit kit (Thermofisher). Patient information is shown in Table S1.

PCR and library preparation

Methylation-independent primers were designed for 23 amplicons (132-257 bp), each positioned in genomic regions hypermethylated in BC and containing >10 CpGs (Table S2). Two multiplex PCRs were performed using 20 ng bisulphite DNA (Zymo lightning), EpiTaq (Takara), 100 nM primers, and 98°C for 30 sec, (98°C for 10 sec, 58°C for 2 min, 72°C for 30 sec) x40, 72°C for 5 min. The PCR products were cleaned with Ampure XP beads (Beckman Coulter) and Nonacus Cell3 adapters were added by ligation following the manufacturer’s protocol. Samples were multiplexed and PE sequenced on a Nextseq 300 cycle mid-output flow-cell to >10,000 average read depth. The training set samples were processed and sequenced on one flow-cell and the test set and additional GB false negatives subsequently processed and sequenced on another flow-cell.

Data analysis

Raw fastq reads were processed using FastQC to check quality metrics. Cutadapt v4.4 was used to trim low-quality data and adapter sequences. Processed reads <30 bases in length were discarded. The quality-checked fastq reads were then mapped to the human genome (version GRCh38) using Bismark Bisulfite mapper v0.24.2 with default settings. The number of methylated and unmethylated reads at each CpG for each sample was extracted from the Bismark alignment output and R v3.6.3 was used for further downstream processing.

Class prediction

The methylation level of each marker was calculated as the median methylation percentage across multiple CpGs. We then calculated the average methylation for each marker across the non-cancer urines in the training set and used mean +3 standard deviations as the threshold for marker positivity and a requirement for at least 3 positive markers for a positive test result. This strategy aimed to maximise the specificity of the methylation test to allow its use as an add-on to GB, potentially increasing a combined test’s true-positive rate, without increasing the false-positive rate.

**

*Figure S1. Average methylation levels across 23 markers and variant allele frequencies of somatic mutations detected by GB. Data = all haematuria patients. Pearson correlation coefficient = 0.77 (p <0.0001).*

*Table S1. Sources of methylation markers. Markers were extracted from the literature or from in-house analysis of TCGA BC versus non-BC methylation array data. Genomic coordinates were then refined using in-house (unpublished) BC WGS with native methylation calling (Oxford Nanopore sequencing) to select CpG rich differentially methylated regions with suitable primer sites.*

| Target | Source |
| --- | --- |
| DMRTA2 | Deng et al 2022 [2] |
| ELAVL4 | TCGA [3] |
| GFRA1 | TCGA [3] |
| GSX1 | TCGA [3] |
| H4C6 | TCGA [3] |
| LINCO1551 | TCGA [3] |
| LINCO1883 | TCGA [3] |
| MEIS1 3' | Beukers et al 2013 [4] |
| MEIS1 5' | Beukers et al 2013 [4] |
| MSC | TCGA [3] |
| ONECUT2 | Beukers et al 2013 [4] |
| OTX1 3' | Beukers et al 2013 [4] |
| OTX1 5' | Beukers et al 2013 [4] |
| OTX2-AS1 | TCGA [3] |
| PAX6-AS1 | TCGA [3] |
| PCDH17 | Costa et al 2011 [5] |
| PCDHGA1 | TCGA [3] |
| PENK | Oh et al 2022 [6] |
| POU4F2 | Reinert et al 2011[7] |
| RYR2 | TCGA [3] |
| SIX6 | TCGA [3] |
| TAFA2 | TCGA [3] |
| ZIC4 | Kandimalla et al 2012 [8] |

*Table S2. Patient Information. N= number of patients per group, N meth+ = number of patients with a positive methylation test result in each group, N SNV+ = number of patients with a positive mutations test (GB) result in each group.*

| Cohort | n | N meth+ | N  SNV+ | Age  (mean, years) | Gender  (F/M) | Stage  (pTa, pT1, pT2+) | Grade  (G1, G2, G3, NK) |
| --- | --- | --- | --- | --- | --- | --- | --- |
| non-BC  (training) | 34 | 2 | 5 | 60.7 | 21/9 | - | - |
| BC  (training) | 30 | 22 | 29 | 70.1 | 5/25 | 20/6/4 | 8/12/9/1 |
| non-BC  (test set) | 28 | 1 | 3 | 51.3 | 14/15 | - | - |
| Haematuria BC  (test set) | 26 | 18 | 24 | 69.8 | 5/21 | 9/10/6  (+1 Ptx) | 1/10/14/1 |
| Additional false negatives | 16 | 0 | 0 | 71.2 | 5/11 | 10/2/4 | 8/2/5/1 |

*Table S3. Average methylation percentages for all methylation markers in all haematuria clinic samples (training and test sets combined). Non-BC ave = average methylation in non-BC urines, BC-ave = average methylation in BC urines.*

| **gene** | **Non-BC**  **ave (%)** | **BC ave**  **(%)** | **Fold-change** | **t-test**  **p-value** |
| --- | --- | --- | --- | --- |
| MEIS1 3' | 16.8 | 55.8 | 3.3 | 6.75E-15 |
| TAFA2 | 8.2 | 48.6 | 5.9 | 1.05E-13 |
| OTX1 5' | 11.5 | 53.6 | 4.7 | 3.5E-13 |
| PCDH17 | 10.7 | 46.0 | 4.3 | 2.95E-12 |
| ONECUT2 | 5.6 | 40.3 | 7.1 | 2.37E-11 |
| OTX1 3' | 7.1 | 33.7 | 4.8 | 1.56E-10 |
| PCDHGA1 | 7.7 | 35.7 | 4.7 | 6.73E-10 |
| RYR2 | 6.7 | 34.1 | 5.1 | 8E-10 |
| LINCO1551 | 7.8 | 37.7 | 4.8 | 1.64E-09 |
| PENK | 4.6 | 21.2 | 4.6 | 8.1E-09 |
| OTX2-AS1 | 10.7 | 34.4 | 3.2 | 9.33E-09 |
| SIX6 | 2.9 | 24.1 | 8.3 | 1.04E-08 |
| GSX1 | 2.6 | 26.0 | 10.1 | 1.38E-08 |
| H4C6 | 2.3 | 32.8 | 14.4 | 1.55E-08 |
| MEIS1 5' | 1.8 | 14.8 | 8.1 | 2.65E-08 |
| PAX6-AS1 | 7.0 | 29.5 | 4.2 | 3.02E-08 |
| GFRA1 | 3.7 | 31.0 | 8.3 | 4.52E-08 |
| POU4F2 | 4.3 | 20.1 | 4.7 | 5.66E-08 |
| MSC | 2.2 | 23.2 | 10.5 | 5.86E-08 |
| LINCO1883 | 2.2 | 27.1 | 12.3 | 1.17E-07 |
| DMRTA2 | 2.0 | 9.0 | 4.4 | 5.16E-07 |
| ELAVL4 | 3.8 | 23.4 | 6.2 | 6.47E-07 |
| ZIC4 | 5.1 | 20.6 | 4.1 | 2.3E-06 |

*Table S4. Methylation markers: Genomic coordinates (GRCh38) and primer sequences.*

**

1. Ward, D., et al., *Highly Sensitive and Specific Detection of Bladder Cancer via Targeted Ultra-deep Sequencing of Urinary DNA.* European Urology Oncology 2023. **6**(1): p. 67-75.

2. Deng, L., et al., *A novel and sensitive DNA methylation marker for the urine-based liquid biopsies to detect bladder cancer.* BMC Cancer, 2022. **22**(1): p. 510

3. Robertson, A., et al., *Comprehensive Molecular Characterization of Muscle-Invasive Bladder Cancer.* Cell, 2017. **171**(3): p. 540-556.

4. Beukers, W., et al., *Hypermethylation of the polycomb group target gene PCDH7 in bladder tumors from patients of all ages.* J Urol. , 2013. **190**(1): p. 311-316.

5. Costa, V., et al., *TCF21 and PCDH17 methylation: An innovative panel of biomarkers for a simultaneous detection of urological cancers.* Epigenetics, 2011. **6**(9): p. 1120-1130.

6. Oh, T., et al., *Identification and validation of methylated PENK gene for early detection of bladder cancer using urine DNA.* BMC Cancer, 2022. **22**(1): p. 1195.

7. Reinert, T., et al., *Comprehensive genome methylation analysis in bladder cancer: identification and validation of novel methylated genes and application of these as urinary tumor markers.* Clin Cancer Res., 2011. **17**(17): p. 5582-5592.

8. Kandimalla, R., et al., *Genome-wide analysis of CpG island methylation in bladder cancer identified TBX2, TBX3, GATA2, and ZIC4 as pTa-specific prognostic markers.* Eur Urol. , 2012. **61**(6): p. 1245-1256.
